# Warm Temperatures Reduce Flower Attractiveness and Bumblebee Foraging

**DOI:** 10.3390/insects12060493

**Published:** 2021-05-25

**Authors:** Charlotte Descamps, Anne Jambrek, Muriel Quinet, Anne-Laure Jacquemart

**Affiliations:** 1 Earth and Life Institute–Agronomy, UCLouvain, Croix du Sud 2, box L7.05.14, 1348 Louvain-la-Neuve, Belgium; annejam2957@gmail.com (A.J.); anne-laure.jacquemart@uclouvain.be (A.-L.J.); 2 Earth and Life Institute–Agronomy, UCLouvain, Croix du Sud 4-5, box L7.07.13, 1348 Louvain-la-Neuve, Belgium; muriel.quinet@uclouvain.be

**Keywords:** bumblebees, floral signals, flower size, nectar, plant–pollinator interactions, pollen, temperature rise

## Abstract

**Simple Summary:**

In the context of climate warming, modifications in plant pollination and reproductive success constitute a crucial issue. Modifications of both floral signals (display, size of flowers) and rewards (nectar and pollen) due to increased air temperatures may affect plant–pollinator interactions. However, relationships between modifications in floral traits and rewards caused by increased air temperatures and the associated effects on pollinator visitation rate and foraging behavior have not been thoroughly investigated. To explore the effects of temperature increase on plant–pollinator interactions, we chose the highly attractive bee-pollinated *Borago officinalis* and one of its pollinators, *Bombus terrestris*. We measured visual floral signals and rewards for plants cultivated at 21 °C or 26 °C and we investigated bumblebee behavior by tracking insect visits on plants in an indoor flight arena. Our results show that exposure to higher temperature during the flowering stages of *B. officinalis* negatively affects visual floral traits (e.g., by reducing the number of flowers) as well as floral rewards, affecting bumblebee visitation and foraging behavior. Bumblebees visited flowers from plants grown at 26 °C four times less frequently than they visited those from plants grown at 21 °C. Thus, the global increases in temperature caused by climate change could reduce plant pollination rates and reproductive success by reducing flower visitation.

**Abstract:**

(1) Background: Plants attract pollinators using several visual signals, mainly involving the display, size, shape, and color of flowers. Each signal is relevant for pollinators foraging for floral rewards, pollen, and nectar. Changes in floral signals and rewards can be induced by an increase in temperature, drought, or other abiotic stresses and are expected to increase as global temperatures rise. In this study, we explored how pollinators respond to modified floral signals and rewards following an increase in temperature; (2) Methods: We tested the effects of warmer temperatures on bee-pollinated starflower (*Borago officinalis,* Boraginaceae) and determined the behavior of one of its main pollinators, the buff-tailed bumblebee (*Bombus terrestris*). We measured visual floral traits (display and size) and rewards (nectar and pollen) for plants cultivated at 21 °C or 26 °C. We investigated bumblebee behavior by tracking insect visits in a binary choice experiment in an indoor flight arena; (3) Results: Plants cultivated at 26 °C exhibited a smaller floral area (i.e., corolla sizes summed for all flowers per plant, 34.4 ± 2.3 cm^2^ versus 71.2 ± 2.7 cm^2^) and a greater flower height (i.e., height of the last inflorescence on the stem, 87 ± 1 cm versus 75 ± 1 cm) compared to plants grown at 21 °C. Nectar production per flower was lower in plants grown at 26 °C than in plants grown at 21 °C (2.67 ± 0.37 µL versus 4.15 ± 0.22 µL), and bumblebees visited flowers from plants grown at 26 °C four times less frequently than they visited those from plants grown at 21 °C; (4) Conclusions: These results show that warmer temperatures affect floral signals and reduce overall floral resources accessible to pollinators. Thus, the global increases in temperature caused by climate change could reduce plant pollination rates and reproductive success by reducing flower visitation.

## 1. Introduction

Declining pollinator abundance and diversity is a major source of concern for biodiversity in the context of global climate change [1,2]. Worldwide, a large proportion of plants depends on pollinators, mostly insects, for their reproduction, and the fraction of crops cultivated for human consumption and needing pollinator activity continues to rise [3,4,5]. Climate change can influence how insects interact with flowers by modifying floral signals and rewards [6,7]. Understanding the processes that strongly affect species behavior in the face of climate change is a major challenge in conservation ecology [8].

Higher air temperatures affect both the vegetative growth and the reproductive development of flowering plants [9,10,11,12,13], with the former generally less sensitive to temperature than the latter. The allocation of resources to reproductive organs may be limited, reducing the production of flowers and affecting flower morphology and fertility [14,15]. However, the temperature at which the plant is subjected to stress is specific to each plant species [10,16,17].

Plants attract pollinators to their flowers using several signals, including flower display, size, shape, color, and odor [18,19,20,21,22,23]. Each signal is relevant for pollinators foraging for floral rewards, pollen, and nectar [24], as pollinators often exhibit innate preferences [25]. Flowers provide floral rewards in exchange for the transfer and deposition of pollen onto pollinators, which promotes reproductive success of pollinated plants [26]. For pollinators, pollen is a rich source of proteins and lipids, while nectar constitutes the main source of sugars [27,28]. Most bees (Anthophila) depend exclusively on these floral rewards for food supply and can adjust their foraging behavior (e.g., moving to a different flower patch) as a function of the quality of available floral rewards [29].

According to the optimal foraging theory [30], pollinators seek to maximize the quantity of food they collect when out foraging, while minimizing their energy expenditure. Flight is energetically costly, and possible rewards from each outing are difficult to predict. The quantity of pollen and nectar varies over time and space [26]. If a flower contains little or no reward, the visit represents a waste of time and energy. Accordingly, pollinators have learned to recognize flowers that offer large rewards [31,32]. Visual signals are particularly decisive in this choice for insect visitors [33]. Pollen and nectar composition (e.g., sugars, amino acids, polypeptides, sterols) determine the nutritional value of floral rewards for pollinators, and high nutritional values are preferred [32,34,35,36,37,38,39].

In the context of global climate change, abiotic stresses such as drought and heat stress are modifying plant–pollinator interactions. For example, pollinators visit flowers from plants suffering from water scarcity less frequently than they visit those of well-watered plants due to the significant decrease in floral visual signals, such as flower number, size, and corolla tube length, in drought-stressed plants [40,41,42]. Increased air temperatures also modify flower signals [17], reducing the number and size of flowers in several entomophilous species [43,44]. Floral rewards also decrease at higher temperature, as measured by a reduction in volume or sugar content of nectar per flower [45,46,47]. In addition, pollen development, and thus fertility and viability, is negatively affected by heat stress [11,48].

Modifications of both floral signals and rewards due to elevated temperatures may affect plant–pollinator interactions, causing morphological and/or recognition mismatches between the two partners [7]. However, relationships between the changes in visual floral traits and floral rewards caused by higher temperatures and the associated effects on pollinator behavior have not been thoroughly investigated. To explore the effects of temperature on pollinator behavior, we chose the highly attractive plant species starflower (*Borago officinalis*) and one of its major pollinators, the buff-tailed bumblebee (*Bombus terrestris*). As visual floral traits and rewards are reduced by higher temperatures in *B. officinalis* [49], we sought to document and characterize the possible changes in visitation and foraging behavior of bumblebees to flowers exposed to a temperature increase of 5 °C. We grew plants at 21 °C or at 26 °C, under otherwise identical controlled conditions in growth chambers. We measured visual floral signals and floral rewards in the two groups of plants. We then presented each group of plants to bumblebees in an indoor flight arena. We investigated bumblebee behavior by tracking insect visits in a binary choice test to flowers from plants grown at 21 °C versus 26 °C. We hypothesized that bumblebees would be less attracted to the flowers of plants grown at 26 °C and visit them less often compared to flowers of plants grown at 21 °C.

## 2. Materials and Methods

### 2.1. Plant Material

Starflower (*B. officinalis*) is an annual, entomophilous plant mainly pollinated by bumblebees and honeybees [50]. Among bumblebees, *B. terrestris* is a frequent visitor of *B. officinalis* [51].

**Figure 1:**
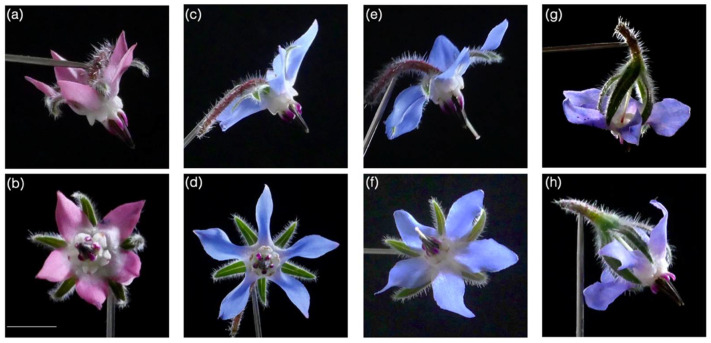
Flower development during anthesis. (**a**,**b**) Stage 1, male phase just after flower opening, with pink or purple petals. (**c**,**d**) Stage 2, transition to female phase with blue petals and stigma inserted. (**e**,**f**) Stage 3, flower with blue petals and protruded receptive stigma. (**g**,**h**) Stage 4, wilting flower before petal abscission. Scale bar, 10 mm.

The flowering period extends from June to September, and about 100 flowers are produced per plant. The flowers, grouped in scorpioid cymes, are hermaphroditic, 5-merous, and protandrous. Their petal color changes from pink to blue during anthesis, which lasts about 3 days. The male phase lasts 1 day, followed by the female phase, which lasts 2 days. Four stages of flower development were distinguished for the experiment (Figure 1).

### 2.2. Insects

We used three colonies of naïve bumblebees, *B. terrestris*, from BioBest Biological Systems (Westerlo, Belgium). Each colony consisted of about 100 workers. The bumblebees had ad libitum access to a nectar solution (Bio-Gluc, BioBest Biological Systems, Westerlo, Belgium), until 2 days before and during the experiments, to motivate them to forage. Polyfloral pollen (Ballot-Flurin, Couteret, France) was also distributed ad libitum.

### 2.3. Experimental Plant Growth Conditions

*B. officinalis* seeds were provided by Semailles (Faulx-les-Tombes, Belgique). Seedlings at the three-leaf stage were transplanted into 2-L pots filled with a 1:1 (*v/v*) mix of sand (size 0/5, M PRO, The Netherlands) and universal peat compost (DCM, Amsterdam, Netherlands) and grown in the greenhouses on the University campus (SEFY platform, Louvain-la-Neuve, Belgium). The plants were watered every 2 days with rainwater.

One hundred plants were cultivated. The two different temperature regimes were imposed on *B. officinalis* plants at floral transition, 7 weeks after sowing, when a flowering stem has developed, and the first floral buds are visible. The plants were subjected to one of two temperature regimes (day/night, 21 °C/19 °C and 26 °C/24 °C) in two growth chambers. Relative humidity was similar in the two growth chambers (80%). The plants were monitored for 4 weeks before experiments with bumblebees. The selection of temperature and stress duration was based on previous study to obtain plants at full boom with visible impact of temperature rise [49].

### 2.4. Visual Floral Trait and Flower Reward Measurements

#### 2.4.1. Visual Signals

To test the effects of a higher growth temperature on plant attractiveness, we measured four floral signals. On observation day, the total number of flowers per plant was counted. Flowers were marked with a water-based marker in order to estimate bumblebee visits per session. After each session of bumblebee visits, all flowers were cut and scanned. We estimated their size by scan analysis using ImageJ software [52]. We calculated floral area per plant, which is the sum of all corolla surfaces per plant, and flower height, defined as the height of the last inflorescence on the main flowering stem.

#### 2.4.2. Floral Resources

Floral resources were measured on five plants per temperature regime, which were not later used for choice experiments with bumblebees. The nectar was extracted with 10-μL glass capillary tubes (Hirschmann Laborgera¨te, Eberstadt, Germany) from 190 flowers (43 flowers at stage 1, 55 flowers at stage 2, 47 flowers at stage 3 and 55 flowers at stage 4). The flower position along the flowering stem and the stage/phase of flower anthesis was recorded. The total sugar concentration (C, g sucrose/100 g solution) was measured with a low-volume hand refractometer (Eclipse handheld refractometer; Bellingham and Stanley, Tunbridge Wells, UK). Nectar sugar content per flower (s, mg) was calculated as s = 10 × d × v × C, where d is the density of a sucrose solution at concentration C (d = 0.0037921 × C + 0.0000178 × C^2^ + 0.9988603) and v is nectar volume (mL) [53]. Pollen production per flower was estimated based on pollen collected from five plants for each temperature regime (total of 66 flowers at stage 2; Figure 1b). The pollen was collected by squeezing and opening anthers with pliers over a microfuge tube. Each pollen sample was weighed to estimate pollen fresh weight (mg) per flower.

### 2.5. Experimental Design of Plant–Insect Interactions

We recorded the behavior of bumblebees visiting plants from the two temperature regime groups. We performed experiments in an indoor flight arena (2.1 m high × 2.8 m long × 2.2 m wide). For each experimental session, we selected ten plants, five plants grown at 21 °C and five plants grown at 26 °C, for 4 weeks before exposure to bumblebees (see 2.3 for plant growing conditions). We arranged plants 40 cm apart to reduce effects from variation in interplant distance on bumblebee movements (Supplementary Figure S1). We carried out nine experimental sessions. The position of the plants (plants grown at 21 °C vs. plants grown 26 °C) was changed between each session to avoid the bumblebees learning the position of the plants. For a same session, all the plants of a same treatment had a similar number of flowers at the same developmental stage. For each plant used for the experimental sessions, we recorded the stage of development of each flower. Since the flowers remain for a very short time in stage 1, and by stage 4 they are almost senescent and fall off the plant, we only considered flowers in stages 2 and 3 for floral resources. We considered the average nectar and pollen quantity for these stages and assigned these values to each flower for the floral resource assessment.

### 2.6. Training Phase and Visiting Observations

Before the experiment, bumblebees were allowed to become accustomed to the flight arena and with the flowers for 3 h. During this training phase, one hive was open, and bumblebees were allowed to move freely.

During each experimental session, we released five bumblebees, one by one, in the flight arena, so that only one bumblebee was present in the flight arena at any given time. One observer noted the visiting sequence by bumblebees and their behavior. Each time that a bumblebee probed a flower was recorded as a visit by the observer. Each visit was timed; the visited plant and the visited flower were also noted. Pollinator feeding behavior was recorded, when possible, as “nectar feeding”, “pollen feeding”, or “nectar and pollen feeding”. More than 3 min was generally necessary for each bumblebee to start visiting flowers. After 20 min of observation, behavior recording was stopped, and the bumblebee captured. Sessions were terminated when bumblebees spent more than 10 min not foraging. All observed bumblebees during each experimental session were replaced in the hive at the end of the session. New plants were used for each session and the position of the plants grown at 21 °C or 26 °C in the flight arena was changed between each session.

### 2.7. Statistical Analyses

Analysis of variance (type I) was performed with a significance level of *p* < 0.05 to evaluate the effects of higher temperature on flower height and on the number of open flowers per plant. Linear mixed models were applied to analyze corolla surface (repeated measurements on the same plant) with one fixed factor (temperature) and plants as the repeated factor. Linear mixed models were used to analyze nectar production (repeated measurements on the same plant) with two fixed factors (temperature and flower developmental stage) and plants as the random factor. Normality of residuals was estimated using QQ plots and homoscedasticity was verified. Tukey’s test was performed for post hoc analyses.

To determine whether bumblebee visit choice was affected by modifications in visual floral traits (flower height, floral area, corolla surface, and number of open flowers) and rewards (nectar volume, concentration, and pollen production), we built a generalized linear model with a binomial distribution. We checked collinearity of the residuals of the model using variance inflation factor (VIF) value (all VIF values were below 5, showing no collinearity between predictors). We used a boxcox transformation (λ = −0.14) for time spent on a flower for data normality to analyze the influence of modifications to visual floral traits and rewards on time spent on a flower. We generated linear mixed models with visual floral traits and rewards as fixed factor and bumblebee individuals as a random factor. Then, we performed χ2 tests to compare feeding behavior on flowers from plants grown at 21 °C (21-flowers) and on those from plants grown at 26 °C (26-flowers). We summarized the data in a two-entry table with temperature (21 °C and 26 °C) and foraging behaviour (nectar, pollen, and nectar + pollen).

We followed the model developed by Ishii [54] to analyze bumblebee visitation sequences. We first excluded bumblebee sequences with less than five visits and took only into account the first ten visits. The frequency of visits to 21-flowers and to 26-flowers was estimated by dividing the number of visits on each flower type by the total number of visits made during the sequence (between five and ten visits). Then, we calculated the proportion of constant flight as the number of constant flights during a sequence (flight from 21-flower to 21-flower or flight from 26-flower to 26-flower) divided by the total number of flights during a sequence (between four and nine, N–1 visits). Ishii [54] defined a coefficient that compares observed and expected numbers of constant flights (C_O/E_). When this parameter is larger than 1, constant flight occurred more often than expected when the sequence of visits is randomly allocated between 21-flowers and 26-flowers.

All analyses were performed in R (version 3.6.1, [55]), using the *car* package for F test, *lme4* package for linear mixed models, *performance* package for collinearity, and *yarr* and *ggplot2* packages for plots. Data are presented as means ± standard errors (SEs) as boxplots and with raw data points included in light gray.

## 3. Results

### 3.1. Effects of Higher Temperature on Plant Attractiveness

#### 3.1.1. Floral Traits

**Figure 2:**
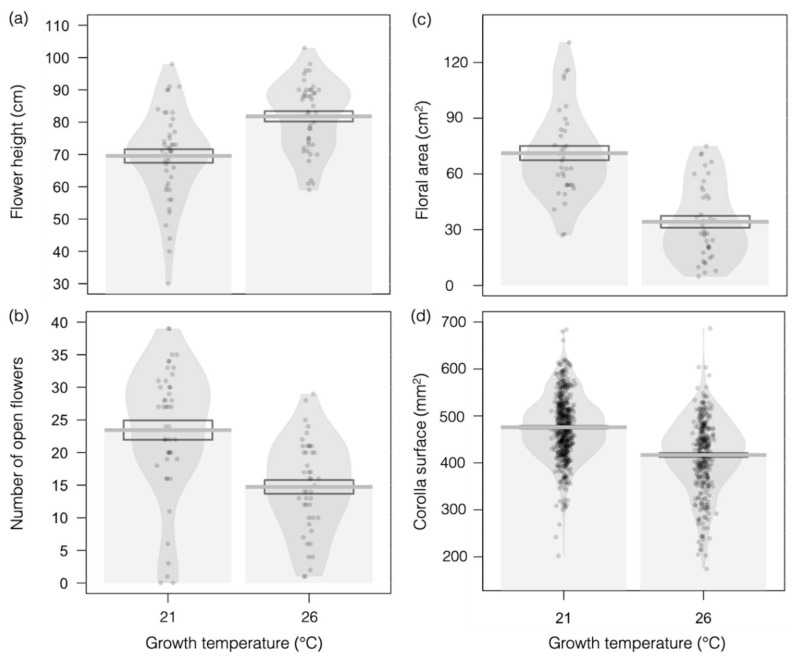
Effects of higher temperature on the visual floral traits of Borago officinalis. (**a**) Flower height (N = 88); (**b**) number of open flowers (N = 88); (**c**) floral area, as defined by the sum of all corolla surfaces per plant (N = 77); and (**d**) corolla surface (N = 909) in plants grown at 21 °C (left-side plots) or 26 °C (right-side plots). Data are presented as means ± standard errors (SEs) as boxplots and with raw data points included in light gray.

Growing plants at higher temperature (26 °C versus 21 °C) induced the elongation of the main flowering stem, as measured by flower height (Figure 2a; *F_1,88_* = 21.48, *p <*0.001) but significantly reduced the number of open flowers per plant (Figure 2b; *F_1,88_* = 22.79, *p <* 0.001), as well as total floral area (i.e., all the corolla surfaces summed up for a plant; Figure 2c; *F_1,77_* = 54.98, *p <* 0.001) and corolla size (Figure 2d; *F_1,909_* = 153.8, *p* < 0.001).

#### 3.1.2. Floral Rewards

**Figure 3:**
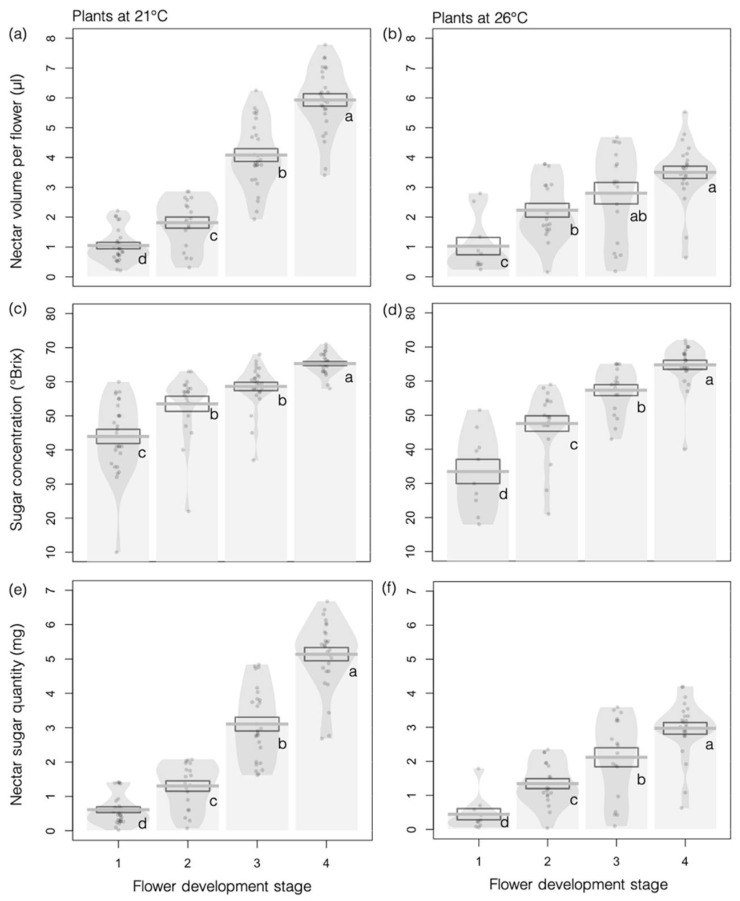
Effects of higher temperature on nectar production in *Borago officinalis*. (**a**,**b**) Nectar volume per flower (N = 178); (**c**,**d**) sugar concentration of nectar; and (**e**,**f**) total amount of nectar sugar (N = 173 for c–f) at the various flower developmental stages of plants grown at 21 °C (**a**,**c**,**e**) or 26 °C (**b**,**d**,**f**). Data are presented as means ± standard errors (SEs) as boxplots and with raw data points included in light gray.

Flowers accumulated nectar throughout their development at both 26 °C and 21 °C, starting at flower opening, as evidenced by increasing nectar volume (Figure 3a; *F_3,154_* = 165.86, *p <* 0.001) and total sugar concentration (Figure 3c; *F_3,157_* = 74.60, *p <* 0.001). Higher temperature negatively affected nectar volume produced per flower during the female phase (Figure 3b; *F_1,13_* = 11.07, *p =* 0.005), although total sugar concentration remained unchanged (Figure 3d; *F_1,13_* = 1.72, *p =* 0.21).

These results indicate that the amount of sugar per flower increased as the flowers developed (Figure 3e; *F_3,154_* = 214.69, *p <* 0.001) but that the increase was less at the higher temperature (Figure 3f; *F_1,13_* = 12.44, *p =* 0.004) during stages 3 and 4 (interaction effect: *F_1,155_* = 16.07, *p <* 0.001).

We observed no significant effects of higher temperature on pollen production (1.02 ± 0.09 mg per flower at 21 °C versus 0.83 ± 0.05 mg per flower at 26 °C; F_1,8_ = 3.67; *p* = 0.09).

### 3.2. Effects of Higher Temperature on Plant–Insect Interactions

**Figure 4:**
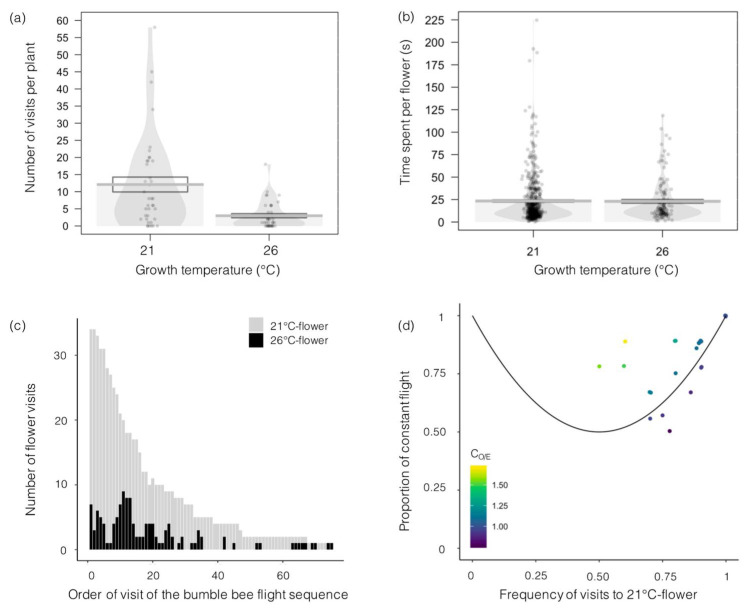
Effects of temperature on bumblebee visits to *Borago officinalis* flowers. (**a**) Number of flowers visited per plant (N = 60); (**b**) time spent per flower visit (N = 653); (**c**) number of flowers of plants grown at 21 °C or 26 °C that were visited according to the order of visit in each flight sequence (sum of all bumblebees flight sequences, N = 34 sequences); and (**d**) proportion of constant flights (from a flower from a plant grown at 21 °C (or 26 °C) to another flower from a plant grown at 21 °C (or 26 °C), divided by the total number of flights) in relation with the proportion of visits to flowers from plants grown at 21 °C. The C_O/E_ coefficient equals 1 along the black line (a sequence above this line, with a C_O/E_ larger than 1, will show more constancy than expected by chance).

We analyzed 34 bumblebee visiting sequences, corresponding to 653 flower visits. We pooled all sequences and discovered that bumblebee individuals visited 518 flowers from plants grown at 21 °C and 135 flowers from plants grown at 26 °C. The number of flowers visited per plant was 4 times greater for plants grown at 21 °C than for plants grown at 26 °C (12 ± 2 versus 3 ± 1 visits per experimental session; Figure 4a).

The choice to visit a flower was significantly predicted by flower height (*χ^2^* = 15.84, df = 875, *p* < 0.001) and floral area (χ^2^ = 8.93, df = 874, *p* = 0.003) but not by corolla surface (*χ^2^* = 2.42, df = 877, *p* = 0.12), the number of flowers per plant (*χ^2^* = 3.27, df = 876, *p* = 0.07), nectar volume (*χ^2^* = 1.33, df = 873, *p* = 0.25), nectar concentration (*χ^2^* = 1.48, df = 872, *p* = 0.22), or pollen production (*χ^2^* = 3.28, df = 871, *p* = 0.07).

Bumblebees, however, visited individual flowers for the same amount of time regardless of plant growth conditions (Figure 4b) and exhibited similar foraging behavior (*χ^2^* = 1.58, df = 2, *p* = 0.45). Indeed, bumblebees visited 19% and 25% of the flowers for nectar foraging, 18% and 21% for pollen foraging and 63% and 54% for both nectar and pollen foraging, on flowers of plants grown at 21 °C and 26 °C, respectively.

Only 6 of 34 bumblebees visited a flower from a plant grown at 26 °C first; all others visited a flower from a plant grown at 21 °C first (Figure 4c). These six individuals then switched to flowers from plants grown at 21 °C for their second and third visits (*data not shown*). Bumblebee visitation sequences showed constancy, especially for flowers from plants grown at 21 °C, as both the proportion of constant flights and the relative frequency of visits to flowers from plants grown at 21 °C were higher than 0.5 (Figure 4d). In most cases (25 of 31), the C_O/E_ was larger than 1 (points were above the 1-C_O/E_ line), indicating higher constancy than expected from a random sequence of visits.

## 4. Discussion

Our results show that exposure to higher temperature during the flowering stages of *B. officinalis* negatively affects visual floral traits as well as floral rewards, affecting bumblebee visitation and foraging behavior.

An increase of 5 °C in plant growth temperature decreased both the total number of flowers per plant and their size, resulting in reduced floral area. Plant phenotypic changes were the consequence of this change in temperature [49,56]. Our results are in agreement with other studies that reported fewer flowers at higher temperature [57,58], although the magnitude of the effect depends on the tolerance of the species (see [59]). Plants grown at 26 °C grew taller (+12%) compared to plants grown at 21 °C in our experiment. Again, at higher temperature, flower height on the main stem can be higher or lower than in plants grown at lower temperature, as a function of plant thermotolerance. Liu et al. [57] observed that a modest (1.5 °C) increase in temperature induced by open top chambers resulted in an increase of flower height for six insect-pollinated species of eight tested. However, a more substantial rise in temperature imposes heat stress and may reduce flower height. For example, exposure of rapeseed (*Brassica napus*) to a temperature of 28 °C for 10 days reduced height by 15% relative to plants maintained at 22 °C [60]. For *B. officinalis*, the higher temperature of 26 °C may initially promote plant growth and drive the observed greater floral height, due to a stimulation of the vegetative growth [49], before limiting later development during flowering, decreasing the number and size of flowers. The optimal temperature for reproductive growth was indeed lower than for vegetative growth in this species [49].

In addition to these modified visual signals, the quantity of floral resources per flower also diminished in response to the 5 °C increase in growth temperature. Higher temperatures impose physiological stress on plants (see [17] and [49] for *B. officinalis)* that may decrease nectar and pollen production per flower [47,61,62]. Nectar secretion is species-specific, and the optimal temperature at which nectar secretion is highest may differ greatly between species [47]. In our experiment, we observed a 50% reduction in nectar volume between plants grown at 26 °C and 21 °C so that the amount of sugar available in nectar per flower decreased with temperature rise. However, pollen production was not significantly affected by the increase in temperature to 26 °C relative to 21 °C under our experimental setup. As the most sensitive stage during pollen grain development is pollen maturation [48], we hypothesize that higher temperatures may negatively influence pollen quality, such as pollen protein content, even if pollen production is quantitatively unchanged. Indeed, we previously observed that temperature rise decreased pollen viability and pollen size in *B. officinalis* [49], suggesting a decrease in pollen quality. Insect visitors can detect such changes in quality and modify their foraging accordingly [24,38], which would reinforce the attractiveness of flowers from plants grown at 21 °C. Despite lower floral rewards per flower at 26 °C, we did not notice any difference in pollinator foraging behavior or time spent foraging per flower. Bees will thus obtain less flower rewards when visiting a flower of plants grown at 26 °C compared to plants grown at 21 °C.

In our experiment, bumblebees foraged on flowers from plants grown at 21 °C with high constancy when given the choice between plants grown at 21 °C or 26 °C. Flowers of plants grown at 21 °C were also more visited and were the first choice of 80% of the bumblebees. Altogether, these results show that bumblebees prefer flowers from plants grown at 21 °C. Flowers from plants grown at 21 °C covered a larger floral area and were placed more compactly along the main stem compared to plants grown at 26 °C. As the floral area is generally considered to be a strong predictor of bee visits [63,64], the smaller floral area at 26 °C may negatively affect the number of visits per plant, which was confirmed by our observations. Rowe et al. [64] suggested that bees use floral area to gauge the nutritional composition of floral resources. Shorter main flowering stems may also reduce the number of visits, since taller plants may be more attractive and visible to pollinators [17,65], although we did not observe more visits to the taller plants grown at 26 °C, which were not particularly more conspicuous than plants grown at 21 °C. Taken together, our results suggest that flowers from plants grown at 26 °C were less attractive to pollinators than flowers from plants grown at 21 °C, even though the changes in signals are contradictory (e.g., a larger floral area enhanced flower attractiveness at 21 °C, although the shorter plants grown at 21ºC are thought to be less attractive). When faced with complex choices with contradictory signals, bees tend to simplify the decision by ignoring some information and focusing on a single signal [66,67].

The cost of foraging (e.g., ratio between energy use and energy intake during foraging, [67]) on plants grown at 26 °C might be higher than that of foraging on plants grown at 21 °C in our experiment as each bumblebee could collect maximum 50% less sugar at 26 °C despite spending the same amount of time per flower. Furthermore, pollinators, especially those depending on flower resources as their sole food source, are threatened when these resources decrease in response to a rise in temperature. The availability of floral resources is a major limiting factor of bee survival in a natural context [28,68].

Plant attractiveness is a complex phenomenon in which several signals converge and interact; while nectar and pollen serve as a reward, the size, shape, smell, and color of a flower serve as an advertisement [33,69,70]. In this study, we took into account only visual signals and rewards, but the choice of visits may also be influenced by other parameters such as flower scent, which may itself be influenced by the higher temperature conditions. Furthermore, foraging decisions result from interactions between innate preference, constancy, choice set composition, cost of foraging and social information, and each of these components interact [67,71,72]. In our experiment, rather than modifying one signal, we opted to study the consequences of changing several floral signals and rewards at once to obtain a more realistic picture of multi-attribute choices compared to oversimplified single-attribute choices that do not reflect reality [67]. However, even if our experimental setup might be more realistic, we remain in an artificial context where insects do not have access to other sources of data such as social information, which are crucial in shaping their decisions.

We observed a strong preference and constancy for 21 °C flowers by bumblebees, based on an analysis of their flight patterns. By changing floral signals and rewards, rising temperatures induced by climate change may have negative consequences for both partners in the plant–insect relationship. Decreasing visitation of plants grown at 26 °C could reduce the frequency of pollen transfer, thus compromising potentially plant reproduction. In addition, lower floral resources or higher foraging cost would have negative nutritional consequences for bees. Despite these negative consequences for both partners, studies on pollinator behavior modifications due to climate change are still rare and should be expanded to other plant–pollinator models.

## Data Availability

The data presented in this study are available on request from the corresponding author.

## Supplementary Materials

The following are available online at https://www.mdpi.com/article/10.3390/insects12060493/s1, Figure S1: Experimental design for bumblebee behavior observations.
