# Supplementary material for: Warm Temperatures Reduce Flower Attractiveness and Bumblebee Foraging
Source: Insects. 2021 May 25;12(6):493. doi: 10.3390/insects12060493 (PMC8226554; doi:10.3390/insects12060493)
Supplement: Supplementary file 1 [file insects-12-00493-s001.zip › insects-1206955-supplementary.pdf]

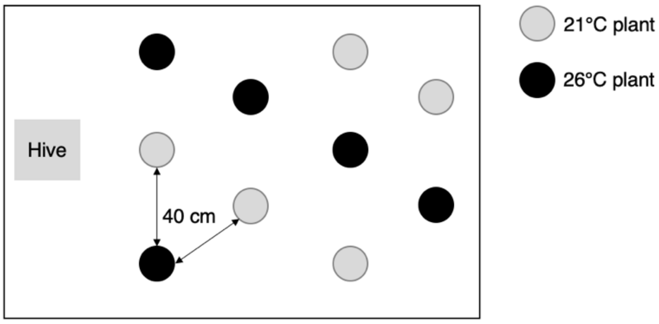

Figure S1. Experimental design for bumblebee behavior observations. The black rectangle represents the flight arena where 10 plants were placed.
